# Kinematic analysis and fault-tolerant trajectory planning of space manipulator under a single joint failure

**DOI:** 10.1186/s40638-016-0048-9

**Published:** 2016-10-03

**Authors:** Zonggao Mu, Liang Han, Wenfu Xu, Bing Li, Bin Liang

**Affiliations:** 1 Shenzhen Graduate School, Harbin Institute of Technology, Shenzhen, 518055 China; 2 Department of Automation, School of Information Science and Technology, Tsinghua University, Beijing, 100084 China

## Abstract

A space manipulator plays an important role in spacecraft capturing, repairing, maintenance, and so on. However, the harsh space environment will cause its joints fail to work. For a non-redundant manipulator, single joint locked failure will cause it to lose one degree of freedom (DOF), hence reducing its movement ability. In this paper, the key problems related to the fault-tolerant including kinematics, workspace, and trajectory planning of a non-redundant space manipulator under single joint failure are handled. First, the analytical inverse kinematics equations are derived for the 5-DOF manipulator formed by locking the failure joint of the original 6-DOF manipulator. Then, the reachable end-effector pose (position and orientation) is determined. Further, we define the missions can be completed by the 5-DOF manipulator. According to the constraints of the on-orbital mission, we determine the grasp envelope required for the end-effector. Combining the manipulability of the manipulator and the performance of its end-effector, a fault tolerance parameter is defined and a planning method is proposed to generate the reasonable trajectory, based on which the 5-DOF manipulator can complete the desired tasks. Finally, typical cases are simulated and the simulation results verify the proposed method.

## Background

SPACE manipulators are expected to be widely used in various space missions, including constructing large space structures, removing orbit debris, and repairing malfunctioned satellite [1–3]. However, a space robot in harsh space environment is prone to failure, resulting in decreased performance of it.

It is extremely difficult, even impossible, to repair or replace these malfunctioning devices on orbit. Therefore, fault tolerance is critical for space manipulators. Because each joint is generally controlled and driven independently, we can deal with the problem respectively when a joint fails. There are mainly two types of joint failure: locked [4, 5] and free-swinging [5, 6] failures. The former refers to a malfunctioning joint that is constrained mechanically, whereas with the latter, actuator torque is lost, and the joint revolves according to the coupling torques of the other joints. In fact, the most common joint failures are joint locked failures. So this paper focuses on the fault-tolerant planning under single joint locked failures. This failure covers two cases: active locking, in which a joint can be locked by fail-safe brakes, and passive locking, in which a joint is locked unexpectedly due to mechanical failure. For previous works [7, 8], the scholars studied the fault-tolerant planning and control methods for redundant manipulators. The redundancy is used to compensate for the motions of the failed joint to continue the designed tasks. However, for a non-redundant manipulator, such as a 6-DOF (degree of freedom) space manipulator, these methods can not be directly applied.

When a joint is locked, a 6-DOF manipulator will decrease to a 5-DOF manipulator, which has insufficient degrees of freedoms to make its end-effector freely move in 3D space. The kinematics, workspace, and trajectory planning are much more different from the original 6-DOF manipulator. There are two types of methods to solve the inverse kinematics of such manipulator with single locked joint: One is to directly solve the 5-DOF manipulator; the other is to construct a new 6-DOF manipulator with a virtual joint. For the former, there are various methods [9, 10] to obtain the inverse solution of the manipulator. The methods based on vector algebra and linear transform [11–13] are always used to solve the inverse solution of a manipulator with insufficient degrees of freedoms. Such manipulator can not attain some given pose (attitude and position), i.e., there will be no any rational inverse solutions for some desired pose. Correspondingly, there will be no rational trajectory to achieve the desired pose. If single variable arc tangent function [14, 15] is used in solving the joint angles, one solution of arc tangent function may be lost.

On the other hand, a virtual joint can be added to construct a new 6-DOF manipulator. Li Xiaotang [16] assumed that the robot grabs bar-like object, so that an attitude parameter corresponding to the gripper rotating around the centerline of rods is not considered. The attitude parameter was regarded as a virtual rotary joint. Then a numerical method was used to solve the inverse kinematics equations. Similarly, Masayuki Shimizu [17] and Zhang Chengkun [18] also constituted a new 6R robot from the 5-DOF manipulator by using a virtual rotary joint. The inverse solution can be derived according to the kinematics of 6-DOF manipulator. However, these papers did not deeply analyze the fault tolerance at any configuration by considering arbitrary joint which is locked.

To take full advantage of space robot under a single joint failure, this paper carries out the workspace analysis for any joint which is fail to work. The bivariate tangent function is used to solve the joint variables, and the quadrant can be determined by the symbolic of independent variable. Then, the analytical inverse solution of the space manipulator under a single joint failure is obtained. By introducing the concept of fault tolerance angle, the 5-DOF robot can complete more tasks at various fault conditions under different fault tolerance requirements. Finally, the Cartesian linear trajectory planning simulation under the 2nd joint failure is performed. The simulation results show the effectiveness of the algorithm.

## Kinematic analysis of a 6-DOF manipulator under single joint failure

### Kinematics modeling and workspace analysis

**Fig. 1:**
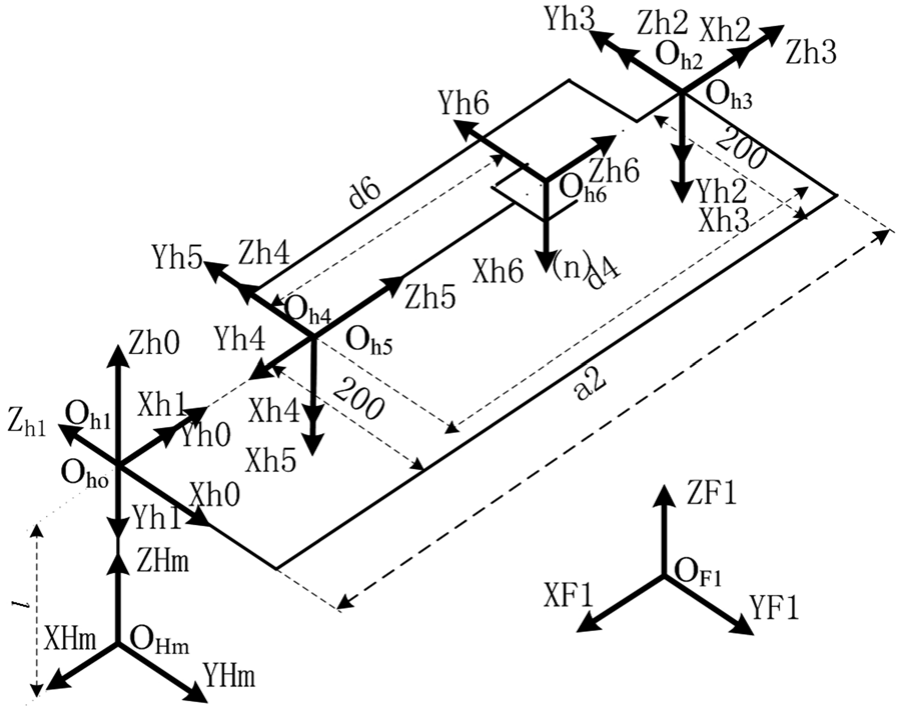
Coordinate system of the manipulator

**Fig. 2:**
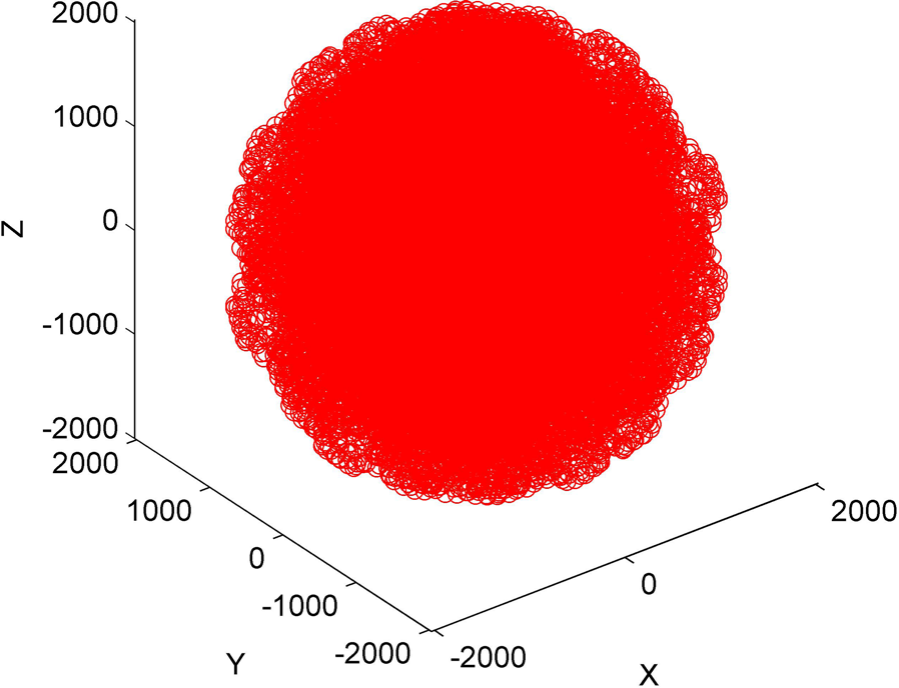
Workspace of normal manipulator

**Fig. 3:**
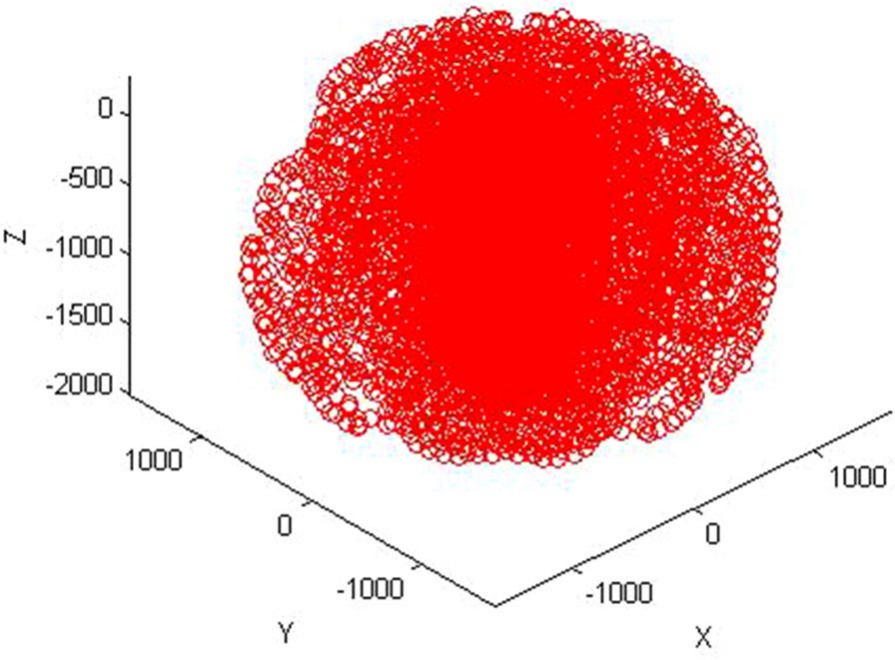
Workspace of failure manipulator (*θ*_2_ = 60°)

According to the structure of the robot, the DH method is used to establish the coordinate system of each link; it is shown in Fig. 1. The corresponding DH parameters are listed in Table 1. Maintop Carlo method is used to obtain the normal workspace which is shown as Fig. 2. Without loss of generality, joint 2 is regarded as the fault joint, and its workspace is shown in Fig. 3; obviously, its workspace becomes a subspace of normal manipulator’s workspace

**Table 1:** D-H parameter of the manipulator

*i*	*a*_*i*−1_/mm	*α*_*i*−1_/°	*d*_*i*_/mm	*θ*_*i*_ */*°
1	0	−90	0	90
2	a_2_	0	0	0
3	0	90	0	90
4	0	−90	*d*_4_	0
5	0	90	0	0
6	0	0	*d*_6_	0

### Inverse kinematics

The main existing inverse solution method for space robot includes analytical method, geometric method, numerical methods and neural network method. Generally, the inverse solution of joint angles is calculated in the range of 360°, and if a single variable arc tangent function is used in solving joint angle, an arc tangent function of the solution may be lost. So the analytical method and bivariate arc tangent function are adopted to solve the inverse solution of space robot in this paper.

### Analytical inverse kinematics of a 5-DOF manipulator

For adjusting the structural parameters of the failure joint and the adjacent joints, the rotation matrix of failure joint is still remained in the kinematic equations, and the current angle of failure joint locked is substituted into kinematic equations. For different failure joints and location, only the corresponding joint variables in the kinematics should be converted to a constant which is currently locked rotation angle. Then the normal analytical solution algorithm is used to solve the equations. This method can improve the solvability of insufficient DOF manipulator and achieve its trajectory planning; this will make sure 6-DOF manipulator still accomplishes most tasks in case of a single joint failure.

If the pose of end-effector has been given and *θ*_2_ is known, the value of *θ*_1_, *θ*_3_, *θ*_4_, *θ*_5_, *θ*_6_ can be determined as follows

1 $$ A_{2}^{ - 1} A_{1}^{ - 1} {}^{0}T_{6} A_{6}^{ - 1} = A_{3} A_{4} A_{5} $$

The corresponding elements (3, 4) in both sides of matrix Eq. (1) are equal:

2 $$ c_{1} (p_{y} - d_{6} a_{y} ) - s_{1} (p_{x} - d_{6} a_{x} ) = 0 $$

The expression of *θ*_1_ can be solved as follows:

3 $$ \theta_{1} = A\tan 2(p_{y} - d_{6} a_{y} ,p_{x} - d_{6} a_{x} ) $$

or,

4 $$ \theta_{1} = A\tan 2( - p_{y} + d_{6} a_{y} , - \,p_{x} + d_{6} a_{x} ) $$

The elements (1, 4), (2, 4) of the left and right sides of (1) are respectively equal:

5 $$ \left\{ {\begin{array}{*{20}c} {d_{4} s_{3} = p_{x} c_{1} c_{2} - d_{6} \left( {a_{x} c_{1} c_{2} - a_{z} s_{2} + a_{y} s_{1} c_{2} } \right) - p_{z} s_{2} - a_{2} + p_{y} s_{1} c_{2} } \\ { - d_{4} c_{3} = d_{6} \left( {a_{z} c_{2} + a_{x} c_{1} s_{2} + a_{y} s_{1} s_{2} } \right) - p_{z} c_{2} - p_{x} c_{1} s_{2} - p_{y} s_{1} s_{2} } \\ \end{array} } \right. $$

$$ {\text{Let:}}\;U = p_{x} c_{1} + p_{y} s_{1} - d_{6} a_{x} c_{1} - d_{6} a_{y} s_{1} , \, V = d_{6} a_{z} - p_{z} $$

The expression of *θ*_3_ can be solved as follows:

6 $$ \theta_{3} = A\tan 2(Uc_{2} + Vs_{2} - a_{2} ,Us_{2} - Vc_{2} ) $$

The left and right sides’ elements (1,3), (2,3) of (1) are respectively equal:

7 $$ \left\{\begin{aligned} c_{3} c_{4} s_{5} + s_{3} c_{5} &= a_{x} c_{1} c_{2} + a_{y} s_{1} c_{2} - a_{z} s_{2} \hfill \\ s_{3} c_{4} s_{5} - c_{3} c_{5} &= - a_{z} c_{2} - a_{x} c_{1} s_{2} - a_{y} s_{1} s_{2} \hfill \\ \end{aligned} \right. $$

$$ {\text{Let:}}h = c_{3} \left[ {a_{z} c_{2} + a_{x} c_{1} s_{2} + a_{y} s_{1} s_{2} } \right] + s_{3} \left[ {a_{x} c_{1} c_{2} - a_{z} s_{2} + a_{y} s_{1} c_{2} } \right] $$

Then the expression of *θ*_5_ can be solved as follows:

8 $$ \theta_{5} = A\tan 2( \pm \sqrt {1 - h^{2} } ,h) $$

9 $$ c_{4} = \frac{{c_{3} }}{{s_{5} }}\left[ {a_{x} c_{1} c_{2} + a_{y} s_{1} c_{2} - a_{z} s_{2} } \right] - \frac{{s_{3} }}{{s_{5} }}\left[ {a_{z} c_{2} + a_{x} c_{1} s_{2} + a_{y} s_{1} s_{2} } \right] $$

The both sides’ elements (3, 3) of (1) are respectively equal:

10 $$ s_{4} = \frac{{a_{y} c_{1} - a_{x} s_{1} }}{{s_{5} }} $$

The expression of *θ*_4_ can be solved as follows:

11 $$ \theta_{4} = A\tan 2\left( {\frac{{a_{y} c_{1} - a_{x} s_{1} }}{{s_{5} }},\frac{{c_{3} }}{{s_{5} }}\left[ {c_{2} (c_{1} a_{x} + s_{1} a_{y} ) - s_{2} a_{z} } \right] - \frac{{s_{3} }}{{s_{5} }}\left[ {s_{2} (c_{1} a_{x} + s_{1} a_{y} ) + c_{2} a_{z} } \right]} \right) $$

The elements (3, 1), (3, 2) of the both sides of (1) are respectively equal:

12 $$ \left\{\begin{aligned} s_{4} c_{5} &= c_{6} ( - n_{x} s_{1} + n_{y} c_{1} ) - s_{6} ( - o_{x} s_{1} + o_{y} c_{1} ) \hfill \\ c_{4} &= c_{6} ( - o_{x} s_{1} + o_{y} c_{1} ) + s_{6} ( - n_{x} s_{1} + n_{y} c_{1} ) \hfill \\ \end{aligned} \right. $$

13 $$ s_{6} = \frac{{c_{4} ( - n_{x} s_{1} + n_{y} c_{1} ) - s_{4} c_{5} ( - o_{x} s_{1} + o_{y} c_{1} )}}{{( - n_{x} s_{1} + n_{y} c_{1} )^{2} + ( - o_{x} s_{1} + o_{y} c_{1} )^{2} }} $$

14 $$ c_{6} = \frac{{s_{4} c_{5} ( - n_{x} s_{1} + n_{y} c_{1} ) + c_{4} ( - o_{x} s_{1} + o_{y} c_{1} )}}{{( - n_{x} s_{1} + n_{y} c_{1} )^{2} + ( - o_{x} s_{1} + o_{y} c_{1} )^{2} }} $$

The expression of *θ*_6_ can be solved as follows:

15 $$ \theta_{6} = A\tan 2(s_{6} ,c_{6} ) $$

It can be seen from the above derivation process of inverse kinematics. When the second joint is failure, *θ*_1_, *θ*_3_, *θ*_4_, *θ*_5_, *θ*_6_ can also be obtained from the formula corresponding to the value of *θ*_2_. Similarly, the analytical inverse solution of other joint failure can also be analyzed.

### Analytical inverse kinematics of a 6-DOF manipulator

In order to avoid the possibility of the solution being lost during the inverse solutions of the manipulator joint angles process, bivariate arctangent function is used in this paper, and then the quadrant of the joint angle can be determined by the argument symbolic. If the pose of end-effector has been given, namely *n*, *o*, *a*, *p* is known, the value of *θ*_1_, *θ*_2_, *θ*_3_, *θ*_4_, *θ*_5_, *θ*_6_ can be determined. This paper gives a solution results directly.

The solutions of joint *θ*_1_, *θ*_2_, *θ*_3_ which determine the position are as follows:

16 $$ \left\{ \begin{aligned} \theta_{1} &= A\tan 2(a_{y} d_{6} - p_{y} ,a_{x} d_{6} - p_{x} ) \, or \, \theta_{1} &= a\tan 2( - a_{y} d_{6} + p_{y} , - a_{x} d_{6} + p_{x} ) \hfill \\ \theta_{2} &= A\tan 2(W, \pm \sqrt {1 - W^{2} } ) - A\tan 2(U,V),\left\{ {\begin{array}{*{20}l} {U = p_{x} c_{1} + p_{y} s_{1} - d_{6} a_{x} c_{1} - d_{6} a_{y} s_{1} } \\ {V = (d_{6} a_{z} - p_{z} )} \\ {W = \frac{1}{{2a_{2} \sqrt {U^{2} + V^{2} } }}\left( {U^{2} + V^{2} + a_{2}^{2} - d_{4}^{2} } \right)} \\ \end{array} } \right. \hfill \\ \theta_{3} &= A\tan 2(Uc_{2} + Vs_{2} - a_{2} ,Us_{2} - Vc_{2} ) \hfill \\ \end{aligned} \right. $$

The solutions of joint *θ*_4_, *θ*_5_, *θ*_6_ which determine the attitude are as follows:

17 $$ \left\{ \begin{aligned} \theta_{5} = A\tan 2( \pm \sqrt {1 - h^{2} } ,h),h = c_{3} [s_{2} (c_{1} a_{x} + s_{1} a_{y} ) + c_{2} a_{z} ] + s_{3} [c_{2} (c_{1} a_{x} + s_{1} a_{y} ) - s_{2} a_{z} ] \hfill \\ \theta_{4} = A\tan 2( - a_{x} s_{1} + a_{y} c_{1} ,\frac{{c_{3} }}{{s_{5} }}\left[ {c_{2} (c_{1} a_{x} + s_{1} a_{y} ) - s_{2} a_{z} } \right] - \frac{{s_{3} }}{{s_{5} }}\left[ {s_{2} (c_{1} a_{x} + s_{1} a_{y} ) + c_{2} a_{z} } \right] \hfill \\ \theta_{6} = A\tan 2\left( {\frac{{c_{4} ( - n_{x} s_{1} + n_{y} c_{1} ) - s_{4} c_{5} ( - o_{x} s_{1} + o_{y} c_{1} )}}{{( - n_{x} s_{1} + n_{y} c_{1} )^{2} + ( - o_{x} s_{1} + o_{y} c_{1} )^{2} }},\frac{{s_{4} c_{5} ( - n_{x} s_{1} + n_{y} c_{1} ) + c_{4} ( - o_{x} s_{1} + o_{y} c_{1} )}}{{( - n_{x} s_{1} + n_{y} c_{1} )^{2} + ( - o_{x} s_{1} + o_{y} c_{1} )^{2} }}} \right) \hfill \\ \end{aligned} \right. $$

## Fault-tolerant trajectory planning methods

### Fault-tolerant planning based on the inverse solution of 5-DOF manipulator

In practice, when a joint of the 6-DOF manipulator fails, the system will lock the joint and keeps it in the current angle. In this way, the 6-DOF manipulator becomes a 5-DOF manipulator, which also names the insufficient DOF manipulator. Actually, it is vital for the insufficient DOF manipulator to complete the expected task accurately by the effective artificially motion control and trajectory planning. For example, a 6-DOF manipulator in aerospace cannot be put to work anymore when a failure happened in one of its joint. But if we use the control system with a new inverse solution algorithm instead of the inverse solution algorithm, in spite of the fact that the manipulator has become the insufficient DOF manipulator, the manipulator can still reach the most pose in its original workspace, and this means that the robot can still put to work and complete the most planning tasks which will improve the capacitive and availability of the entire spacecraft system.

When there is a single joint failure in a 6-DOF manipulator, its related workspace will be reduced accordingly, as shown in Fig. 3. Therefore, the manipulator is still able to complete part of its task based on its ability to work. As for the tasks that a 5-DOF manipulator with the fault can complete, we can carry out the trajectory planning with conventional planning methods, including getting the inverse solution in accordance with “Analytical inverse kinematics of a 5-DOF manipulator” of formula 5-DOF and calculate the angles about the movable joint.

### Fault-tolerant planning based on the inverse solution of 6-DOF manipulator

#### The introduction of fault-tolerant method

The insufficient DOF manipulator can only reach part of the position and attitude in their original workspace, so that the inverse solution of the desired position and attitude may not exist. Because not all the location and attitude are solvable, the unrealizable attitudes of manipulator will lead to the failure of expected tasks. In most cases, the vector algebra, linear transformation, and other methods are used to get the inverse solution about the position of the insufficient DOF manipulator. But if there is no inverse solution of a certain posture when using the ordinary inverse solution algorithm, the manipulator trajectory planning cannot be achieved and the insufficient DOF manipulator with a failure joint will not able to complete the task. Therefore, it is full of high research value and practical value to study inverse solution algorithm about the position of 6-DOF manipulator with fault-tolerant performance.

**Fig. 4:**
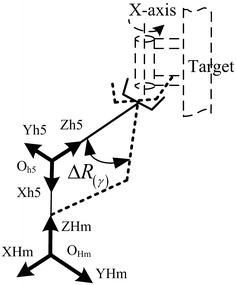
Gripper rotates around the centerline (X-axis) Δ*R*_(γ)_

When the manipulator intends to grab the rod or other similar objects, we will not restrict the attitude parameters if the gripper rotates around bar’s centerline. Then the rotation variables can be regarded as a virtual unknown rotary joint which is shown in Fig. 4, so inverse position problem of the insufficient DOF manipulator can be converted into an inverse solution problem. Due to the change in structural parameters and the uncertainty of the joints and location of the fault, the inverse solution program must meet the requirements of the uncertain fault state. In this paper, we traversed all the value of the rotation angle around the X-axis and obtained overall relationship diagram between the rotation angle around X-axis and the corresponding joint with a failure in order to select the proper angle for the planning values.

The algorithmic process is as follows:

1. When a single joint fails, the desired joint angle of each joint can still be substituted into the kinematic equations in order to calculate the target point relative to the base coordinate system of the position and attitude matrix_*e*_^0^*T*;
2. The matrix_*e*_^0^*T* is multiplied with the homogeneous transformation matrix of the rotational angle *γ* which rotates around X-axis of the tool coordinate system, we obtain _*e*_^0^*T*:
  18 $$ {}_{e}^{0} T \cdot \left[ {\begin{array}{*{20}c} 0 & {} & \quad 0 &\quad 0 &\quad 0 \\ 0 &\quad {\cos (\gamma )} & {} &\quad { - \sin (\gamma )} & 0 \\ 0 & \quad{ \, \sin (\gamma )} & {} &\quad {\cos (\gamma )} & 0 \\ 0 &\quad {} & 0 &\quad 0 &\quad 1 \\ \end{array} } \right] $$
3. Respectively, the matrix _*e*_^0^*T*^′^ is calculated corresponding to the increments of *γ* from −180° to 180° and the inverse kinematics solution of the manipulator is obtained based on _*e*_^0^*T*^′^;
4. In the condition that the type of reference manipulator has been set, the value of the joint angle can be determined one to one using the inverse kinematics solution according to the failure joint when the angle *γ* changes in the range of 360°. So we can draw the diagram between the attitude angle *γ* and the fault joint.
5. The values of each joint angle that meet the job requirements can be obtained based on a consideration of the range of fault-tolerant angle, real fault angle of the fault joints.
6. After determining each desired angle, the trajectory will be re-planned in accordance with the analytic solution of inverse kinematics of the 5-DOF to complete scheduled tasks.

The algorithm takes advantage of the normal arm inverse kinematics, so that we don’t need to re-identify the parameters and reconstruction of the manipulator even when the joint fails. After obtaining the graph, we can consider the actual fault condition about the fault tolerance and the size of angle in the fault joint. As a result, when the target matrix rotates around the X-axis in coordinate system with the angle *γ*, the insufficient DOF manipulator can still be able to complete the scheduled tasks due to the substitute motion by other joints. But if a joint failure occurs in some less flexible operating space, the value of angle *γ* calculated by this method will be relatively large, which leads to the fact that the manipulator will fail to complete scheduled tasks with the solution of the six joint angle values, because the corresponding position and posture of the manipulator may cause a collision between the end-effector and the target object.

### The analysis of fault tolerance under a single joint failure

**Fig. 5:**
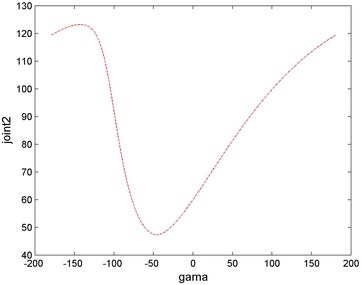
Overall relationship between the angle rotating about the X-axis and joint 2 diagram

**Fig. 6:**
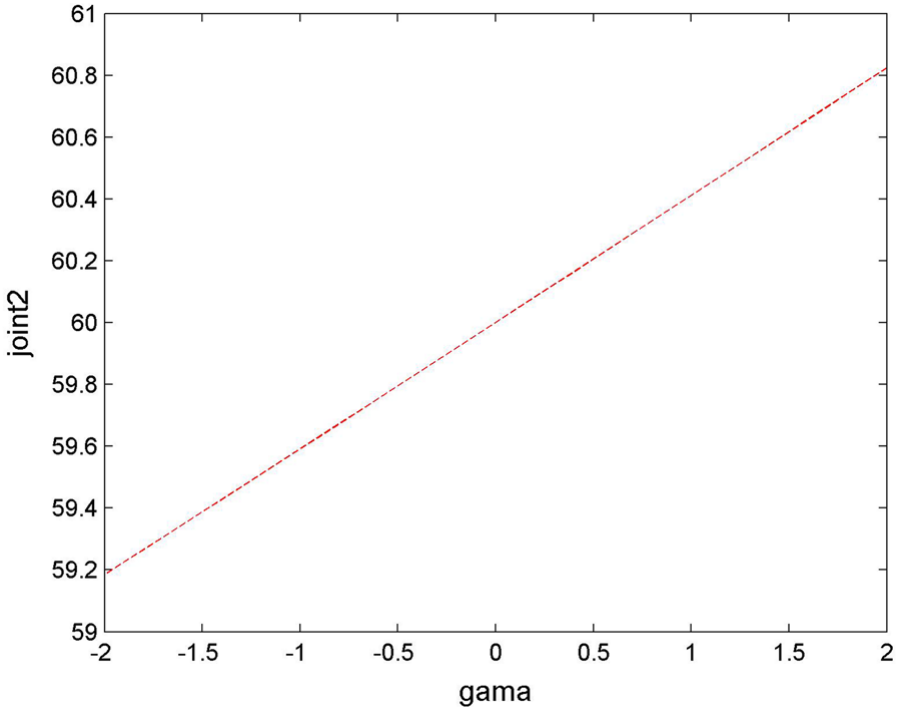
Relationship between joint 2 and the rotation angle *γ*

We can carry out the work about the mission planning under the condition that the fault angle of joint 2 is 60°. Also we set the initial movement joint *q*0 = [0, 60, 0, 0, 0, 0]°, while the terminate movement joint: *q*f = [5, 60, 35, 10, −120, 90]°; the initial speed: *qv*0 = [0, 0, 0, 0, 0, 0]; the termination rate: *qv*f = [0, 0, 0, 0, 0, 0]; from Fig. 5, it can be seen that when the rotation angle rotating around the X-axis changes in the range of ±180°, fault compensation range of joint 2 is 48° to 123°. As shown in Fig. 6, when the angle which is the movement attitude of the end-effector rotates about the X-axis, the failure compensation range of the joint 2 is 59.2° to 60.8°.

**Fig. 7:**
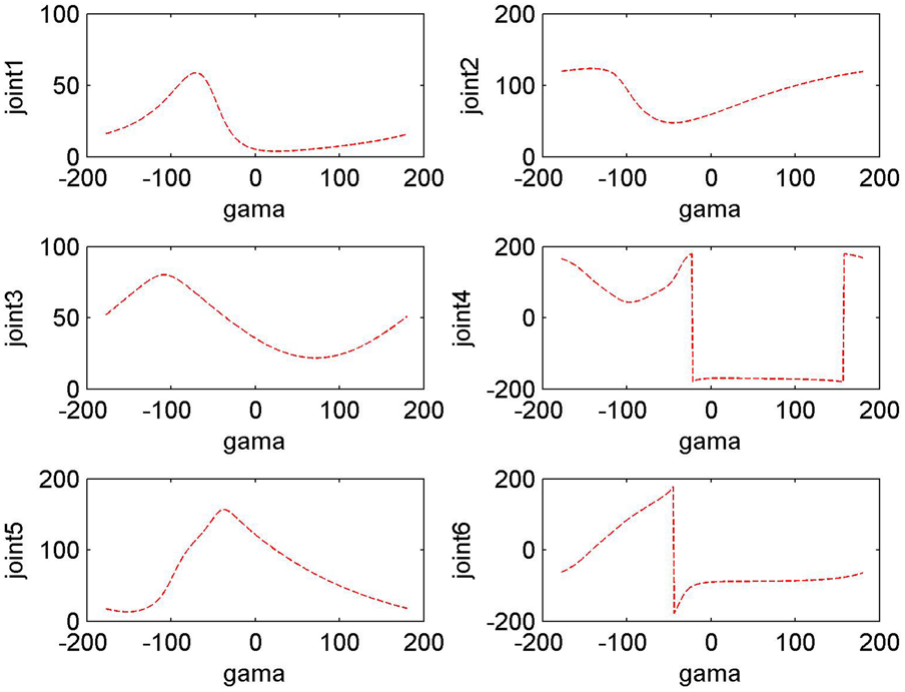
Overall relationship between joint 1–joint 6 and the rotation angle *γ*

**Fig. 8:**
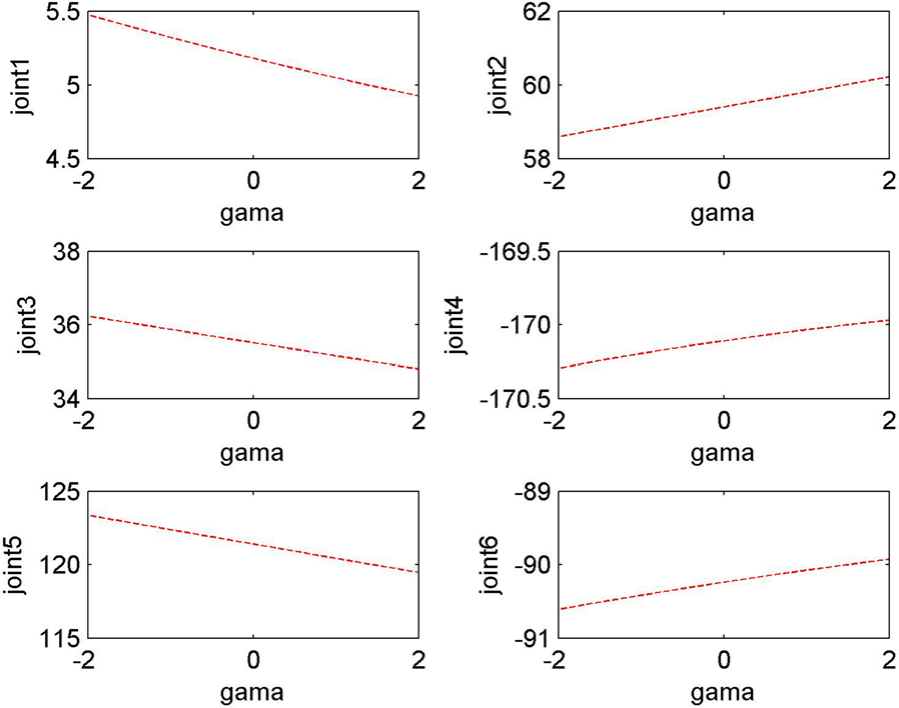
Relationship between joint 1–joint 6 and the rotation angle *γ*

It is assumed that a failure occurs in the second joint of the space robot when its angle is 59.4° and we can get *γ* = −1.45 according to the fault-tolerant algorithm. At this point the six joint angle values obtained for the target point and performing the tasks are *q*f = [5.18, 59.40, 35.51, 9.89, −121.40, 89.76]°. It means that when the failure occurs in the second joint, in order to approach the target, the space robot will need other joints’ substitute motion. When the end-effector reaches the desired position, the rotation angle will be equal to −1.45° in this case. Figures 7 and 8 show the fault tolerance range of joint 1–joint 6 when *γ* is in [−180°, +180°] and [−2°, +2°], respectively.

## The trajectory planning and simulation about fault tolerance under the typical tasks

**Fig. 9:**
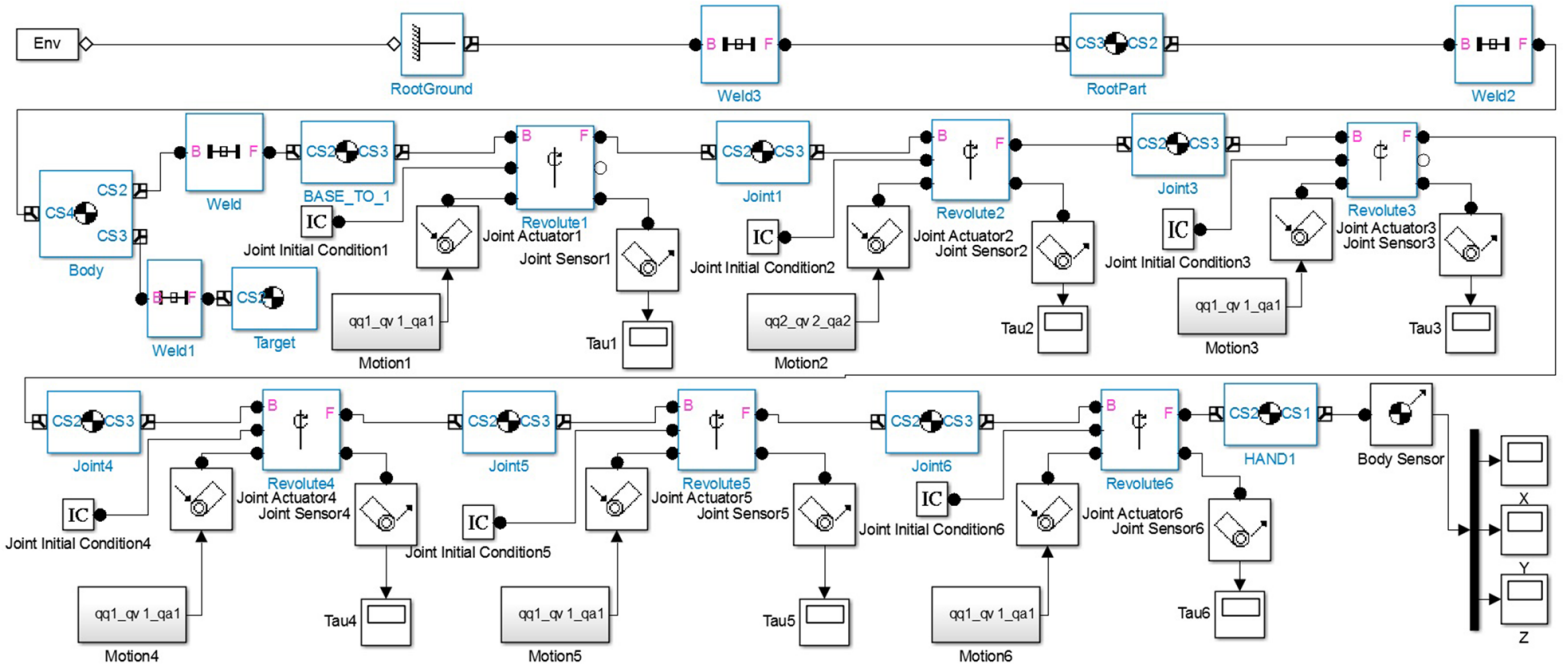
Simulation system

Based on the Simulink, the SimMechanics is an interdisciplinary research and analysis environment for the controller and the target system. Acting as an intuitive and effective modeling and analysis tools for the multi-body dynamic mechanical systems and control systems, the SimMechanics completes all its work in the Simulink environment. In this paper, ProE5.0 and MatlabR2010b jointly establish a simulation systems as shown in Fig. 9, so as to carry out simulation. Taking the linear motion in Cartesian space for example, if we know the starts and ends coordinates A(*x*_*a*_, *y*_*a*_, *z*_*a*_),C(*x*_*c*_, *y*_*c*_, *z*_*c*_) of the line in the space and the interpolation number *N*, then we get:

19 $$ \left\{ {\begin{array}{*{20}c} {\Delta x = (x_{c} - x_{a} )/(N + 1)} \\ {\Delta y = (y_{c} - y_{a} )/(N + 1)} \\ {\Delta z = (z_{c} - z_{a} )/(N + 1)} \\ \end{array} } \right. $$

For any point *i* (1 ≤ *i*≤ *N*) on the line, we have

20 $$ \left\{ {\begin{array}{*{20}c} {x_{i} = x_{a} + \Delta x \cdot i} \\ {y_{i} = y_{a} + \Delta y \cdot i} \\ {z_{i} = z_{a} + \Delta z \cdot i} \\ \end{array} } \right. $$

Based on the discrete end straight path in the formulas (19) and (20), we can carry out verification of the fault-tolerance planning under the single joint failure.

### The straight trajectory planning under conditions of the failure in joint 2 (without fault-tolerant attitude error)

**Fig. 10:**
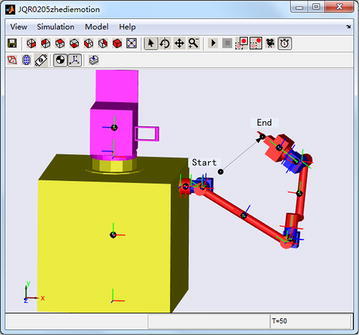
Trajectory of the end-effector

**Fig. 11:**
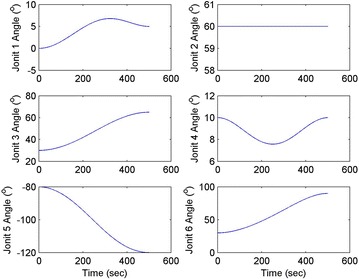
Angle variation of each joint

**Fig. 12:**
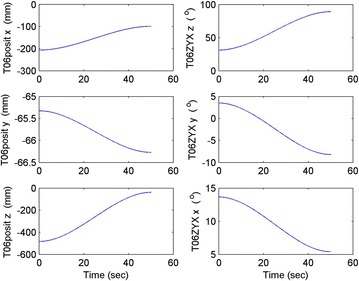
Change in the attitude and position of the end-effector

**Fig. 13:**
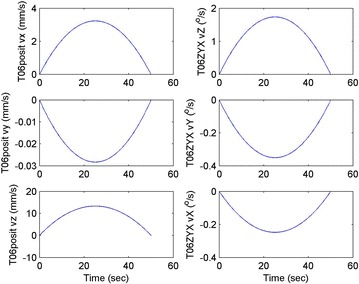
Error of the attitude and position of the end-effector

**Fig. 14:**
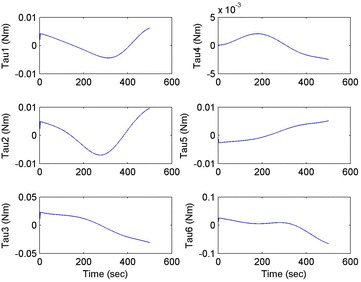
Change in the attitude and position of the end-effector

**Fig. 15:**
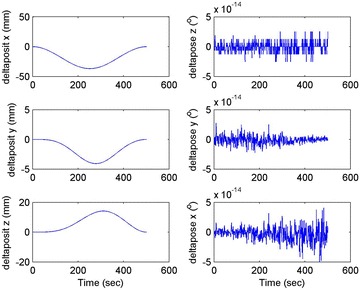
Control force of each joint

Initial position: [−206.16, −65.32, −482.55], the initial attitude Euler angles: [0.54, 0.06, 0.23]. Terminal position: [−98.33, −66.26, −37.81], terminal Euler attitude angle: [1.55, −0.14, 0.09], according to the inverse kinematics of each joint in the paper, we can get the initial joint angle of each joint *q*_0 = [0, 60, 30, 10, −80, 30] and the terminal joint angle *q*_f = [5, 60, 65, 10, −120, 90]. The initial and terminal states are shown in this section in Fig. 10. The planning cycling position of the linear motion is 250 ms. The angle variation of each joint is shown in Fig. 11. The change in the attitude and position of the end-effector is shown in Fig. 12. Therefore, we can get the tracking error of the end position that is less than 2° from Fig. 13, which means that the position eventually reaches our expectation. The speed of the attitude and position of the end-effector are shown in Fig. 14. Applying the PD control to each joint according to the linear motion in the Cartesian space of the end-effector, we obtain the control force of each joint as shown in Fig. 15.

### The straight trajectory planning under conditions of the failure in joint 2 (fault-tolerant attitude error ≤10°)

Initial position: [62.7315, −65.3258, −520.9870], the initial attitude Euler angles: [0.5931, −0.3826, −0.0452]. Terminal position: [−98.3390, −66.2696, −37.8197], terminal Euler attitude angle: [1.5573, −0.1427, 0.0945], according to the inverse kinematics of each joint in the paper, we can get the initial joint angle of each joint *q*_0 = [0, 30, 30, 10, −80, 30] and the terminal joint angle *q*_f = [5, 60, 65, 10, −120, 90].

We carry out the task planning of the fault tolerance in the condition that the joint 2 fails (similar to the situation in other joints) when the manipulator is working if it cannot achieve the desired angle of 60°. When the joint fails at 52.0547°, as we can see in Fig. 5, the posture tolerance that the end-effector rotates around the X-axis is −10°. So the actual posture can be obtained:

21 $$ T06 = \left[ {\begin{array}{*{20}c} {0.0134} &\quad { - 0.9966} &\quad { - 0.0819} &\quad { - 98.3390} \\ {0.9897} &\quad {0.0249} & \quad { - 0.1407} & \quad { - 66.2696} \\ {0.1422} &\quad { - 0.0792} &\quad {0.9867} &\quad { - 37.8197} \\ 0 &\quad 0 & \quad 0 &\quad 1 \\ \end{array} } \right] $$

After the introduction of fault tolerance angle, the actual configuration of manipulator can be determined at this time. This indicates that when the manipulator has not yet reached the desired second joint of 60°, it is necessary for the other joints to do some replacement motion so that the end-effector of the robot can get closed to the target ultimately and work with the posture that the original desired position rotates around the X-axis with −10°. By the inverse solution we can obtain:

22 $$ qq \, = \, \left[ {10.5789 \, 52.0547 \, 66.1915 \, 8.5610 \, - 124.0978 \, 82.7611} \right] $$

With the fault tolerance angle, we get: end position [−98.3390, −66.2696, −37.8197] and terminal attitude Euler angles: [1.5573, −0.1427, −0.0801]. According to the actual location of the fault of the joint, we can re-plan the end of the gesture to carry out the task in the case of without affecting the arrest. By solving it can be found that when the joints fail at 52.0547°, the fault tolerance angle is −10°, which can be substituted by the movement through other joints, and the manipulator still is able to complete the task.

**Fig. 16:**
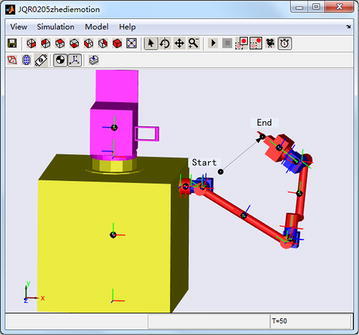
Trajectory of the end-effector

**Fig. 17:**
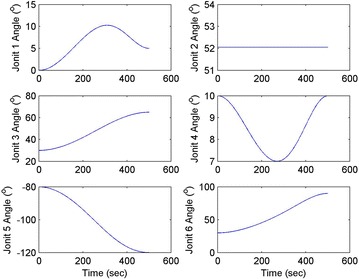
Angle variation of each joint

**Fig. 18:**
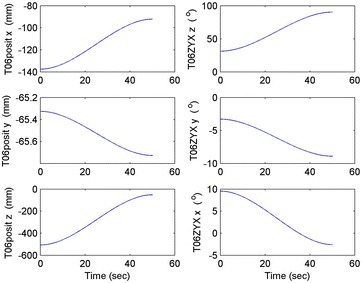
Change in the attitude and position of the end-effector

**Fig. 19:**
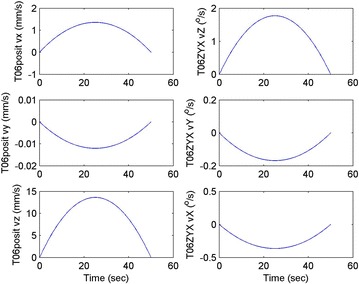
Error of the attitude and position of the end-effector

**Fig. 20:**
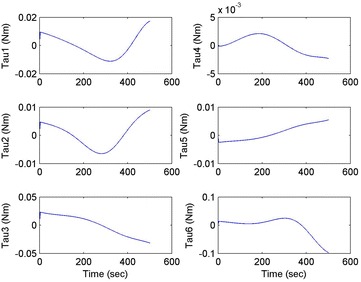
Change in the attitude and position of the end-effector

**Fig. 21:**
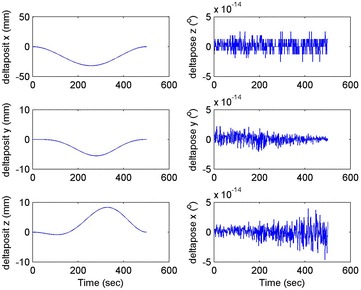
Control force of each joint

The initial and final states are as shown in this section in Fig. 16. Linear motion planning cycling position is 250 ms. The angle variation of each joint is shown in Fig. 17. The change in the attitude and position of the end-effector is shown in Fig. 18. Therefore, we can get the tracking error of the end position that is less than 2° from Fig. 19, which means that the position eventually reaches our expectation. The speed of the attitude and position of the end-effector is shown in Fig. 20. Applying the PD control to each joint according to the linear motion in the Cartesian space of the end-effector, we obtain the control force of each joint as shown in Fig. 21.

## Conclusion

This paper conducts a research on fault tolerance trajectory planning for a 6-DOF space manipulator under a single joint failure, and the workspace of the manipulator for pre- or post-failure is analyzed based on the actual situation of joint failure. The analytical inverse kinematics equation of the 5-DOF formed from the 6-DOF manipulator with locking single joint is derived to ensure the completion of part tasks in the workspace. In order to further represent the ability of the manipulator under a single joint failure, the concept of fault-tolerant angle is introduced. Then the relationship between the fault tolerance angle and the locked joint is established. Based on it, the best choice of desired angle of each joint which is most suitable for specific task can be determined. This paper provided an effective way to maximize the ability of a space manipulator under a single joint failure.
